# Implantable Shock Absorber for Knee Osteoarthritis in Older Patients: Evidence From Two Clinical Studies

**DOI:** 10.7759/cureus.112880

**Published:** 2026-07-17

**Authors:** Dennis Crawford, David C Flanigan, Seth Sherman, Jacek Walawski, Claude Moorman, Thomas Carter, Christina Carlson, Anil Ranawat, Konrad Slynarski, David Diduch

**Affiliations:** 1 Orthopaedics and Rehabilitation, Oregon Health & Science University School of Medicine, Portland, USA; 2 Orthopaedic Surgery, Ohio State University Wexner Medical Center, Columbus, USA; 3 Orthopaedic Surgery, Stanford University, Redwood City, USA; 4 Orthopaedics, Zagiel Med Hospital, Lublin, POL; 5 Orthopaedics, Atrium Health, Charlotte, USA; 6 Orthopedic Surgery, University of Arizona College of Medicine - Phoenix, Phoenix, USA; 7 Orthopaedics, Oregon Health & Science University, Portland, USA; 8 Orthopaedics, Hospital for Special Surgery, New York, USA; 9 Orthopaedics, LEKMED, Warsaw, POL; 10 Orthopaedics, University of Virginia, Charlottesville, USA

**Keywords:** clinical study, implantable shock absorber, knee osteoarthritis (koa), older adults, pain and function outcomes

## Abstract

Background

Knee osteoarthritis (OA) is a common, chronic condition associated with significant pain and functional limitations. Patients who receive inadequate relief from conservative care and are not indicated for or are unwilling to undergo arthroplasty procedures have previously faced a significant treatment gap. To address this, we examined the implantable shock absorber (ISA) in a cohort of patients over 65 years old, at follow-up through five years.

Methods

Patients from two clinical studies (Atlas and Calypso) aged 65 and over during the five-year observation period were evaluated in this secondary analysis. Primary outcomes included pain and functional ability (measured via the Western Ontario and McMaster Universities Arthritis Index (WOMAC)). Secondary outcomes included return-to-activities and adverse events. Outcomes were collected at baseline and at routine follow-up assessments for five years post ISA treatment. Follow-up measurements are compared with baseline at each interval.

Results

A total of 18 participants (12 females) were eligible for inclusion in the analysis (mean age at final follow-up: 68.0 years, mean body mass index (BMI): 28.1). Five years after treatment with ISA, pain scores had improved by 70% over baseline (mean of 17.1 (SD 19.3) vs. mean of 55.0 (SD 11.9); p<0.01), and function scores had improved by 64% (mean of 19.5 (SD 22.2) vs. mean of 53.4 (SD 17.0); p<0.01). Additionally, 13/14 (93%) of participants achieved a clinically meaningful improvement in pain, with an 11/14 (79%) incidence of functional improvement after five years. Patients reported limitations in the performance of desired physical activities at baseline; at five-year follow-up, 14/18 (77.8%) of participants were able to perform one activity with no or minimal limitations (p<0.0001 vs. baseline). One device malfunction was reported at 105 days post procedure. Three participants were elected for conversion to total knee arthroplasty (TKA) in the study period.

Conclusions

Our study demonstrates that the ISA is a viable intervention for a selected population of patients with knee OA aged 65 and older. In this particular population, ISA was associated with significant long-term and sustained improvements in pain and function without compromising joint integrity, thus preserving options for future arthroplasty. For patients suffering in the treatment gap between the limited effectiveness of non-surgical approaches and the finality of arthroplasty, treatment with ISA offers a viable and effective proven option.

## Introduction

Osteoarthritis (OA) is a chronic, progressive disease affecting over 350 million patients worldwide [1], including over 32 million in the United States (US) alone [2]. With 29.5 million incident cases annually, OA represents one of the leading causes of disability worldwide [1]. Knee and hip OA are recognized as leading causes of mobility impairment in older adults in the US [3], with knee OA alone associated with healthcare costs in excess of $72 billion annually [4]. OA affects a broad spectrum of patients - 13.9% of adults over age 25 have clinical OA in at least one joint [3]; however, it disproportionately impacts older adults, with those aged 65 and over accounting for 33.6% of OA diagnoses [2,3].

Treatment options for knee OA are many and often take a multimodal, stepwise approach, with "conservative" treatments, such as weight loss, pain management, mobility maintenance, and viscosupplementation, typically recommended as first-line therapies [5-8]. While variably effective in managing symptoms in early stages, these treatments do not provide long-term relief as the condition advances and health consequences deteriorate [9,10]. As this occurs, some patients transition from the non-invasive therapies to surgical options, such as arthroscopic debridement or high-tibial osteotomy (HTO), although disease progression ultimately leads many patients to partial or total knee arthroplasty (TKA) [11,12]. The challenge for patients and surgeons alike is that this progression is rarely linear, leaving many individuals to suffer through a period where they are failing non-operative care yet do not meet the criteria for, or do not intend to undergo, knee replacement. This familiar "treatment gap" historically leaves patients with few options, creating frustration and potentially amplifying quality of life-related and general health concerns [5,13-15]. This is especially true of patients 65 and over, who are less likely to benefit from arthroscopic surgery [16-18] and who may be unwilling to undergo arthroplasty [19]. Treatment options other than arthroplasty also enhance shared decision making and thus improve patient satisfaction [20,21].

Interventions for patients in this treatment desert are historically limited. Now, a technology applying joint protection, the implantable shock absorber (ISA), is designed to meet this need and relieve the symptoms of medial knee OA through a minimally invasive surgical procedure. This novel technology, in use clinically since 2008, unloads the medial compartment of the knee, thereby reducing pain while preserving both motion and joint integrity. In doing so, it offers a complementary treatment option for patients who are non-responsive to non-operative care and are unwilling to undergo or ineligible for TKA. The ISA has been the subject of multiple clinical studies demonstrating robust safety and effectiveness, as well as favorable pain and function outcomes compared with HTO [22,23]. The majority of evidence, however, is from patients under age 65. Despite the high prevalence of OA and the absence of reimbursement barriers in older adults in the US, evidence in this population remains limited, underscoring additional challenges in this treatment gap.

With this background in mind, we sought to examine the ISA in an older adult population with knee OA. Our objective was to leverage data from two existing clinical studies and perform a secondary analysis of data in patients aged 65 or older during the five-year study observation period. Our hypothesis was that, at five years follow-up, those 65 and over would see significant and clinically meaningful improvements in pain and function scores over baseline, as well as restoration of desired activity five years after ISA treatment.

## Materials and methods

Study design

Two prospective, open-label cohort studies of the ISA in adult patients with knee OA (Atlas, NCT02934659; Calypso, NCT03671213, NCT03838978) were completed and recorded in accordance with routine regulatory processes. Studies were conducted in a total of 15 centers across the US (n=10) and Europe (n=5), with 17 surgeon investigators enrolling 131 participants (Calypso, n=81; Atlas, n=50). This manuscript reports a secondary analysis of patients from the Atlas and Calypso trials who were aged 65 and over during the five-year follow-up, reflecting a clinically and policy-relevant population defined by age-based health coverage eligibility for patients in the US.

Study participants

The eligibility criteria for both studies have been outlined in detail elsewhere [22,23] and are summarized in full in Table 1. In brief, Atlas and Calypso enrolled patients aged 25 years and older with radiographic evidence of OA in the medial compartment of the knee (Kellgren-Lawrence (KL) grades 1-4 [24]) and failure of ≥ 6 months of non-surgical treatment. Further eligibility criteria included a BMI of ≤ 35 or < 300 lbs and a baseline Western Ontario and McMaster University Osteoarthritis Index (WOMAC) pain score of ≥ 40. Patients with lateral OA (> KL 1) or advanced patellofemoral OA (≥ KL 3), major contralateral disease, or recent joint-modifying surgery were excluded. Participants were censored at the time of surgical conversion to TKA. For this secondary analysis, only patients aged 65 years or older at a follow-up visit during either trial were included. At baseline, participants from each trial did not differ significantly in mean age (Atlas: 65.1 (SD: 6.5) vs. Calypso: 61.6 (1.6), p=0.21), mean KL grade (2.9 (1.0) vs. 2.5 (0.9), p=0.41) or mean BMI (28.0 (3.5) vs. 28.1 (4.0), p=0.96), facilitating combining of the study populations.

**Table 1:** Eligibility criteria for the Atlas and Calypso clinical studies AAOS: American Academy of Orthopaedic Surgeons; BMI: body mass index; DEXA: dual-energy X-ray absorptiometry; IA: intra-articular; KL: Kellgren-Lawrence; KOOS: Knee Osteoarthritis Outcomes Survey; WOMAC: Western Ontario and McMaster University Osteoarthritis Index Source: Refs [22,23]

Variable	Atlas	Calypso
Trial title/designation	Atlas Knee System Clinical System Clinical Study (USA) (Atlas-USA)	Calypso Knee System Clinical Study
	NCT02934659	NCT03671213, NCT03838978
Eligibility criteria		
Inclusion criteria	Male or female subjects age 25-80 years at time of screening	Subjects age 25-65 years at time of screening
	Body mass index (BMI) of < 35 or weight < 300 lbs	Body mass index (BMI) of < 35 and weight < 300 lbs
	Has pain in the study knee as demonstrated by a minimum score of 40 (scale 0-100) on the WOMAC pain questions in KOOS	WOMAC pain ≥ 40
	Knee flexion ≥ 90°	Knee flexion ≥ 90° and ≤ 140°
	Has failed at least six (6) months of non-operative treatment with continued osteoarthritis (OA) pain. Prior treatment is defined as treatment with at least one of the following interventions (listed on the AAOS Clinical Practice Guidelines on the Treatment of Osteoarthritis of the knee non-arthroplasty): lifestyle modification, Weight loss, if BMI ≥ 35, pain relievers, physical therapy, orthotics (splints, braces), intra-articular (IA) corticosteroid injections	Failed at least 6 months of non-operative treatment defined as at least one of the treatments per AAOS treatment of osteoarthritis of the knee; evidence-based guideline 2nd edition 2013
	Clinical symptoms (such as pain primarily localized to the medial aspect of the knee and generally exacerbated by weight bearing) and radiographic evidence of osteoarthritis in the medial compartment of the knee. Weight-bearing Fixed Flexion view is recommended to verify radiographic evidence of osteoarthritis. KL grade 1-4, except those with bony erosion	Clinical symptoms (such as pain primarily localized to the medial aspect of the study knee and generally exacerbated by weight bearing) and radiographic evidence, using a weight-bearing fixed flexion view, of osteoarthritis in the study knee as defined as KL grade of 1-4
Exclusion criteria	Clinical symptoms or radiographic evidence of osteoarthritis in the lateral compartment of the study knee defined as Kellgren & Lawrence (KL) grade of > 1	Clinical symptoms or radiographic evidence of osteoarthritis in the: Lateral compartment of the study knee defined as KL grade of > 2, Patellofemoral compartment of the study knee defined as KL grade > 3
	Clinical symptoms or radiographic evidence of osteoarthritis in the patella-femoral compartment of the study knee defined as KL grade ≥ 3	Radiographic evidence of large, defined as > Grade 2+, marginal osteophytes in the medial compartment of the study knee or bony erosion
	Clinical symptoms or radiographic evidence of osteoarthritis at the contralateral knee that would preclude activity of daily living, stair climbing, stair descending, or requires the use of an assist device	Clinical symptoms or radiographic evidence of osteoarthritis at the contralateral knee that would preclude activity of daily living, stair climbing, stair descending, or requires the use of an assist device
	Tibial-femoral alignment of more than 10° of varus, or more than 6° of valgus, as measured using anatomical axis on a standing Hip-Knee-Ankle or long standing AP view X-ray OR Hip-Knee-Ankle alignment of more than 16° of varus, or more than 0⁰ of valgus, as measured using mechanical axis on a standing Hip-Knee-Ankle AP view x-ray	Mechanical axis alignment (hip-knee-ankle) < -16° (varus) or > 0° (valgus) OR anatomic axis alignment < -10° (varus) or > 6° (valgus) as measured on a long standing AP X-ray view
	Previous lateral meniscectomy >30% of the study knee, previous patellar surgery in the study knee, or previous osteotomy or failed knee joint replacement in the study knee	Previous knee surgeries: Lateral meniscectomy, patellar surgery, osteotomy, prior knee implant, or knee joint replacement of the study knee
	Previous joint modifying surgery in the study knee within 12 months prior to planned surgery date such as ligament reconstruction, meniscus repair, cartilage transplantation, and microfracture	Joint modifying surgery in the study knee within 12 months of index procedure (e.g., ligament reconstruction, meniscus repair, cartilage transplantation, microfracture, etc.)
	Arthroscopic surgeries for joint lavage, meniscectomy, chondral debridement, and loose body removal are excluded if within 3 months prior to planned surgery date	Arthroscopic surgeries in the study knee within 3 months of index procedure for joint lavage, meniscectomy, chondral debridement, and loose body removal
	Hyperextension >5 ° and Flexion contracture > 10°	Hyperextension > 5° and Flexion contracture > 10°
	Pathologic ligamentous or meniscal instability (Lachman > 1) as assessed by the investigator	Pathologic ligamentous (Lachman > 1) or meniscal instability
	Suspected or documented allergy or hypersensitivity to cobalt, chromium, iron, or nickel metals	Allergy or hypersensitivity to cobalt, chromium, iron, or nickel metals (suspected or documented)
	Active infection, sepsis, osteomyelitis or history of septic arthritis in any joint	Active infection, sepsis, osteomyelitis or history of septic arthritis in any joint
	Rheumatoid arthritis, other forms of inflammatory joint disease or autoimmune disorder	Rheumatoid arthritis, other forms of inflammatory joint disease or autoimmune disorder
	Paget's disease or metabolic disorders which may impair bone formation	Paget’s disease or metabolic disorders which may impair bone formation
	Known or suspected diagnosis of osteomalacia, known or suspected diagnosis of osteonecrosis	Osteomalacia or osteonecrosis (known or suspected)
	Osteoporosis or radiolucency of the femoral or tibial cortex on x-ray suggestive of moderate to severe osteoporosis, pathologic fractures, or bone mineral density T score of > 2.5 standard deviation (SD) below young adult reference mean (all subjects will be screened for risk of osteoporosis using the validated Osteoporosis Self-Assessment Tool (OST) score, subjects with high risk will undergo a DEXA scan to determine eligibility	OST score of < 4 for women or < 6 for men AND a T-score less than -1.0 based on a DEXA scan of the proximal femur (preferred) or lumbar spine. All subjects with an OST score < 4 for women or < 6 for men will undergo a DEXA scan to determine the bone mineral density T-score
	Charcot joint disease or other severe neurosensory deficits	Charcot joint disease
	Rapid joint destruction, marked bone loss or bone resorption apparent on X-ray	Known or suspected rapidly destructive OA (RDO) as determined by the investigator
	Vascular insufficiency, muscular atrophy, neuromuscular disease	Insufficiency of the cruciate and collateral ligaments, which would preclude stability of the Calypso Knee System
	History of systemic steroid treatment, medication use that affects bone metabolism (such as chemotherapy) within the previous 6 months, or radiotherapy within the previous 6 months	Steroid treatment (oral or IV), medication use that affects bone metabolism (such as chemotherapy) or radiotherapy within 6 months of index procedure
	Immunologically suppressed or immunocompromised	Bone malignancy (active or history)
	Any significant medical condition	Co-morbidities that could impact study participation or results
	Subjects who are currently involved in any investigational drug or device trial or have been enrolled in such trials within the last 3 months	Subjects who are currently involved in any investigational drug or device trial or have been enrolled in such trials within 3 months of index procedure
	Other factors that the investigator feels would interfere with the participation and completion of the study	Other factors that the investigator feels would interfere with the participation and completion of the study

Study outcomes and follow-up

Primary and secondary outcomes for the Atlas and Calypso trials are summarized in Table 2. In both studies, the primary outcome was a composite measure of clinical success at 24 months post procedure, defined primarily as a minimum 10-point and 20% improvement from baseline in WOMAC pain and function scores, freedom from device-related serious adverse events (SAEs), preservation of implant integrity as assessed radiographically, and freedom from conversion to arthroplasty or HTO. Atlas additionally required maintenance of the normal joint range of motion as part of its primary outcome.

**Table 2:** Outcome measures for the Atlas and Calypso clinical studies HTO: high tibial osteotomy; KOOS: Knee Osteoarthritis Outcomes Survey; KSS: Knee Society Score; OARSI: Osteoarthritis Research Society International; UCLA: University of California, Los Angeles; WOMAC: Western Ontario and McMaster University Osteoarthritis Index; WORMS: whole-organ magnetic resonance imaging score Source: Refs [22,23]

Variable	Atlas	Calypso
Primary outcome(s)	24-month composite endpoint individual subject success demonstrating non-inferiority. A subject was declared a clinical success if all of the following components were met at the 24-month follow-up.	24-month composite endpoint demonstrating non-inferiority of the Calypso Knee System to HTO data. A subject was a responder if all of the following components were met at 24 months
	Clinically significant improvement of at least 20% from baseline on the WOMAC pain questions in the KOOS questionnaire with a change of ≥ 10 points	Clinically significant improvement of at least 20% from baseline on the WOMAC pain questions in the KOOS Knee questionnaire with a change of ≥ 10 points
	Clinically significant improvement of at least 20% from baseline on the WOMAC function questions in the KOOS questionnaire with a change of ≥ 10 points	Clinically significant improvement of at least 20% from baseline on the WOMAC function questions in the KOOS Knee questionnaire with a change of ≥ 10 points
	No subsequent surgical intervention of the medial knee (including device failures requiring removal or revision). No serious device-related adverse events	Freedom from the following device-related serious adverse events (SAEs): deep infection requiring surgical intervention (both arms), damage to adjacent neurovascular or ligament structures necessitating reconstruction (both arms), non-union (HTO only). Endpoint Subsequent Surgical Intervention (SSI)
	Maintenance of implant integrity as evaluated by radiographic assessment. Implant integrity will be assessed following the Atlas Image Evaluation Protocol	Maintenance of implant integrity as evaluated by radiographic assessment
	Maintenance of normal range of motion (ROM) defined as: Knee flexion ≥ 90 degrees. Knee extension within 10 degrees of the "neutral" or zero degree position	
Secondary outcome(s)	Clinical outcomes will be determined using the following outcome measurement tools to evaluate symptom severity changes at each follow up visit compared to baseline: Knee injury and Osteoarthritis Outcome Score (KOOS), including WOMAC pain, function, and stiffness; Knee Society Scores (KSS), Investigator Knee Function Examination; Knee Society Scores (KSS), Subject-Administered Questionnaire; UCLA Activity Score, Subject-Administered Questionnaire; Return to Work; Knee range of motion measured passively defined as degrees of flexion and extension	Secondary endpoints demonstrating superiority of the Calypso Knee System to HTO data for each of the following: Recovery: time to full weight bearing. Early WOMAC pain and function defined as percent change from baseline to 3 months on the WOMAC pain in the Knee injury and Osteoarthritis Outcome Score (KOOS Knee Survey) and Percent change from baseline to 3 months on the WOMAC function in the knee injury and Osteoarthritis Outcome Score (KOOS Knee Survey). Durability of WOMAC pain and function defined as Percent change from baseline to 24 months on the WOMAC pain in the Knee injury and Osteoarthritis Outcome Score (KOOS Knee Survey) and Percent change from baseline to 24 months on the WOMAC function in the Knee injury and Osteoarthritis Outcome Score (KOOS Knee Survey)
Safety Observations	Safety outcomes will be determined by evaluating the type, frequency, severity, and relatedness of adverse events through the 24 month time point for all subjects	Knee range of motion (ROM), measured passively defined as degrees of flexion and extension. Adverse events by type, over time, severity, seriousness, and relatedness. Secondary surgical interventions by type of surgical intervention (revision, removal, re-operation (includes conversion to arthroplasty) supplemental fixation, other intervention). Metal ion levels. In the event of a Calypso Knee System removal, radiographic evaluations, MRI assessment as available, Calypso Knee System (implant) retrieval analysis, histology, metal Ion levels, arthroplasty conversion data, if applicable
Effectiveness/Other Observations	Radiographic and MR images will be evaluated using the following measurements: Atlas Knee Implant integrity; Kellgren & Lawrence OA classification; OARSI; WORMS	Patient reported knee outcome measures and activity (KOOS Knee Survey, KSS, Subject Activity and Satisfaction, SF-12 Health Survey). Investigator reported KSS

Secondary outcomes collected in Calypso included the return-to-activities, time to weight bearing, and the documentation of adverse events. For return-to-activities, each participant completed a physical activity questionnaire modeled on the Discretionary Knee Activities portion of the Knee Society Score [25]. Participants chose three activities (yielding a total of 54 activity selections) and rated their ability to perform each activity using the following categories: Cannot Do, Major Limitation, Somewhat Limited, Minimal Limitation, or No Limitation. Adverse events were assessed at each follow-up visit, with SAEs adjudicated by an independent medical monitoring board. Outcome assessments were conducted at baseline and at six weeks, three and six months, and 1, 1.5, 2, 3, 4, and 5 years post procedure.

For this secondary analysis, we examined the change from baseline in WOMAC pain and function scores from eligible Atlas and Calypso participants as the primary outcome. Secondary outcomes included return-to-activities, the occurrence of adverse events, and implant integrity as evaluated through radiographic analysis.

Radiographic analysis

Plain film radiography was obtained at each follow-up visit, and implant integrity was assessed via independent adjudication as either (a) implant intact or (b) loss of implant integrity (disassembly or component breakage).

Surgical technique

The full surgical technique for the implantation of the ISA (MISHA Knee System; Moximed, Inc., Fremont, CA) has been detailed [22,23,26-28]. In brief, the ISA is implanted with the patient in a supine position using a medial approach and aligned with the medial collateral ligament (MCL). An incision proximal to the femoral condyle and extending to the skin above the pes anserine exposes the medial soft tissue and the MCL fibers. Dissection and retraction expose the sub-vastus pocket for femoral component placement. Tibial and femoral landmarks are identified, and a trial instrument is used to orient the implant for alignment and clearance through a full range of motion. The final implant is secured with six locking screws, and soft tissue closure is completed without tension. Procedures are completed on an outpatient basis. The studies did not involve concomitant procedures, and participants were instructed to engage in immediate weight bearing as tolerated and a full range of motion without restriction postoperatively.

Statistical analysis

Descriptive statistics are presented as mean and standard deviation (SD) (or range, X-Y) for continuous variables or as number (n or N) with percentage for categorical variables. Mean values were compared using a paired Student's t-test. while proportions were compared using the chi-squared test or Fisher's exact test, as appropriate. Statistical significance was defined a priori as p<0.05 for all statistical comparisons. Patients achieving a clinically meaningful improvement in WOMAC pain and function scores of ≥ 10 points and 20% change from baseline were classified as responders [29,30].

## Results

Study participants

**Figure 1:**
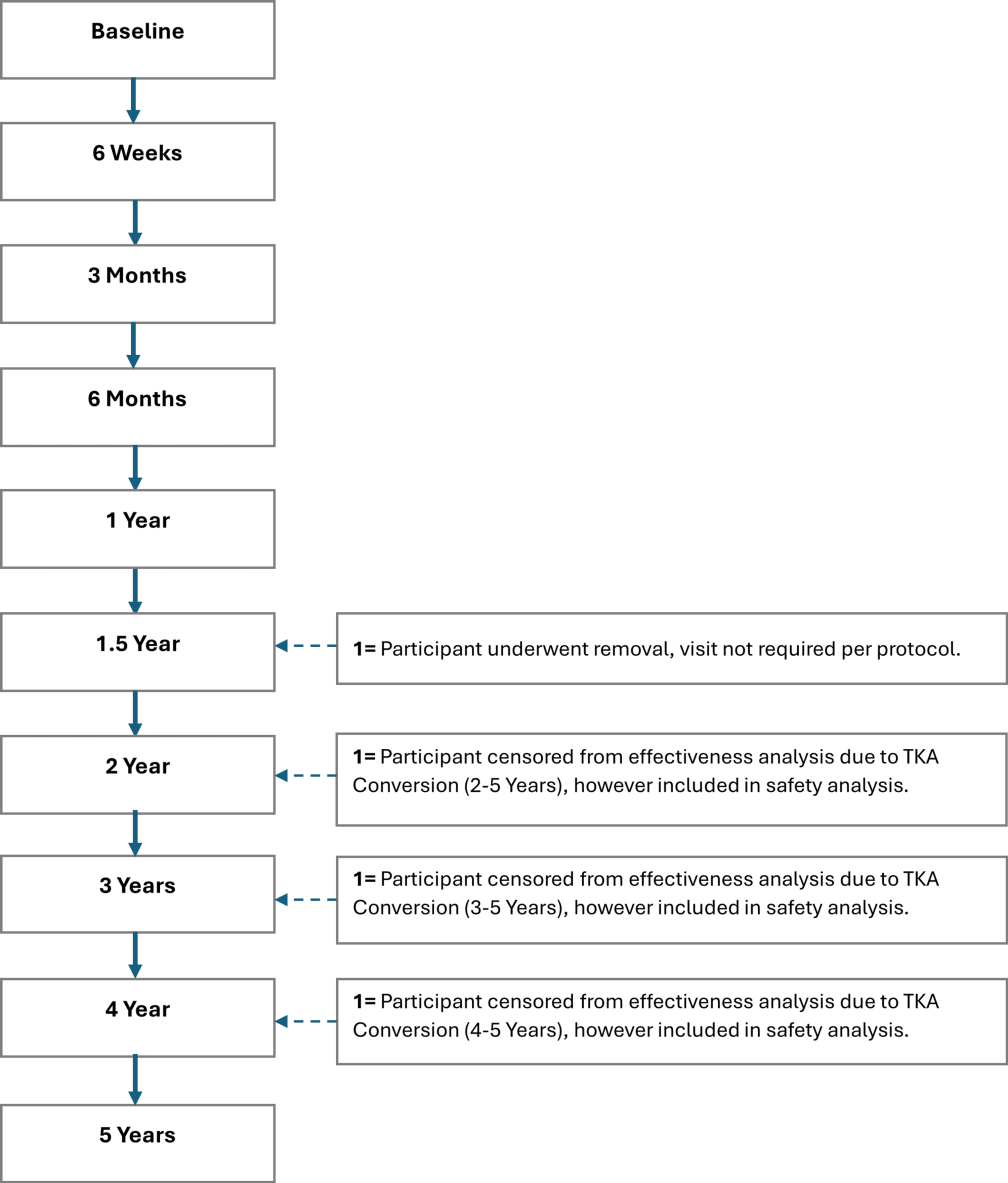
Participant accountability TKA: total knee arthroplasty

A total of 18 participants from Atlas (n=7) and Calypso (n=11) met the inclusion criteria and were included in this analysis (Figure 1). Full demographic details are summarized in Table 3. The mean age for included participants at five years post-procedure was 68.0 years (range: 65-80 years). A total of 53 measures of effectiveness in patients were recorded and analyzed over five years, with a patient mean of 3.5 measures, and the primary outcome change from baseline at five years post treatment. At baseline, participants had a mean BMI of 28.1 (3.7) and a mean KL grade of 3.2. Females constituted 12/18 (67%) of the included population.

**Table 3:** Summary of demographic statistics for the combined study cohort BMI: body mass index; KL: Kellgren-Lawrence

Variable	Study cohort (N=18)	Range (%)
Age (years), mean (range) 0, index procedure	63	60-75
Age (years), mean (range) - 5-year follow-up	68	65-80
Sex	N	%
Male	6	33
Female	12	67
BMI (kg/m^2^), mean (range)	28.1	22.1-34.7
KL grade, mean (range)	3.2	2-4
Race	N	%
White	18	100
Black	0	0
Ethnicity	N	%
Hispanic or Latino	1	5.6
Not Hispanic or Latino	17	94.4
Tobacco use	N	%
Yes	6	33.3
No	12	66.7
Operative knee	N	%
Left	12	66.7
Right	6	33.3

Study outcomes

WOMAC Pain

**Figure 2:**
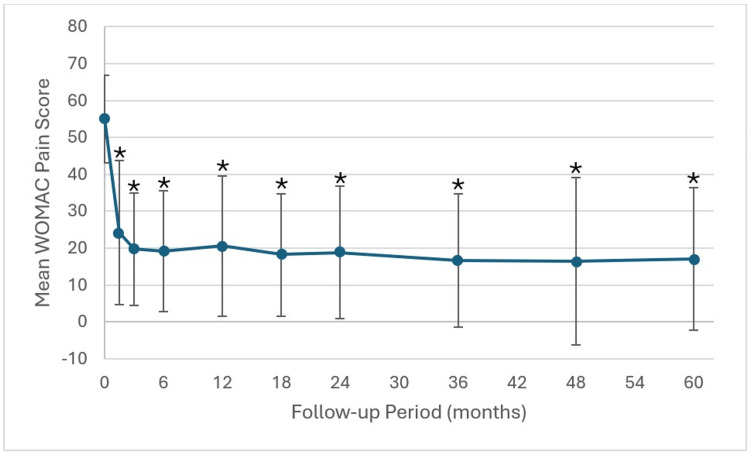
Mean WOMAC pain score change over time * indicates statistically significant difference vs. baseline (p<0.05). Results available for 18 participants to 12 months, 17 participants at 18 and 24 months, 15 participants at 36 months, and 14 participants at 48 and 60 months. WOMAC: Western Ontario and McMaster University Osteoarthritis Index

WOMAC pain scores were available for 18/18 patients up to the one-year follow-up visit and for 14/18 participants at the five-year follow-up visit. Pain scores improved significantly postoperatively, with six-week follow-up scores (24.2 (19.5)) significantly decreased from baseline (55.0 (11.9), p<0.0001). Similar improvements were noted at the two-year (18.8 (17.9), p<0.0001 vs. baseline) follow-up. At five years, pain scores had decreased by 70% to a mean of 17.1 (SD 19.3) (p<0.0001 vs. baseline), with a mean change from baseline of -35.7 (SD 15.4) points (Figure 2). The responder rate at five years was 13/14 (93%) participants who had achieved a clinically meaningful improvement in pain scores. 

WOMAC Function

**Figure 3:**
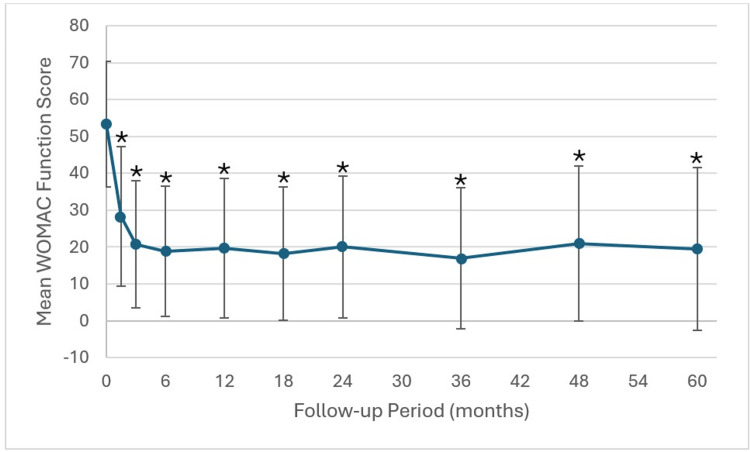
Mean WOMAC function score change over time * indicates statistically significant difference vs. baseline (p<0.05). Results available for 18 participants to 12 months, 17 participants at 18 and 24 months, 15 participants at 36 months, and 14 participants at 48 and 60 months. WOMAC: Western Ontario and McMaster University Osteoarthritis Index

WOMAC function scores were available for 18/18 participants up to the one-year follow-up and for 14/18 participants at the five-year follow-up visit. At baseline, the mean score was 53.4 (17.0), which improved significantly by the six-week follow-up visit (28.3 (19.0), p=0.0002). At two-years post procedure, function scores remained significantly improved (20.0 (19.3), p<0.0001 vs. baseline), and by the five-year follow-up, function scores had improved by 64% to a mean of 19.5 (SD 22.2) (p<0.0001 vs. baseline) with a mean change from baseline of -30.6 (SD 21.5) points (Figure 3). At the five-year follow-up, 11/14 (79%) participants met the threshold for a clinically meaningful improvement.

Return to Activity

**Figure 4:**
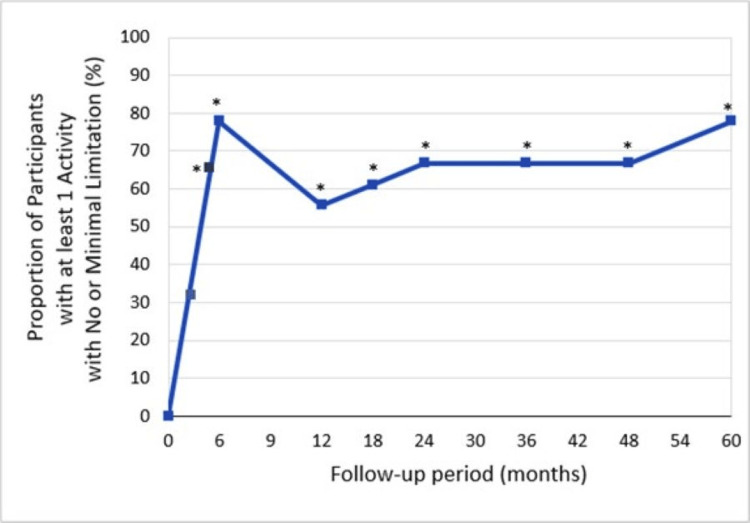
Participant ability to perform the desired physical activities, including the proportion of participants able to perform at least one activity with Minimal or No Limitations at each follow-up * indicates statistically significant difference vs. baseline (Fisher's exact test, p<0.05).

The most common desired activity among participants was running/jogging/hiking/distance walking, 13/54 (24.1%), followed by cycling/stationary bike/elliptical use, 11/54 (20.4%). Baseline assessment found that all activities in all participants were performed with limitation (i.e., no activity was rated as performed with Minimal or No Limitation). At five years, 14/18 (77.8%) participants were able to perform at least one of their desired activities with Minimal or No Limitation. Six weeks after ISA treatment, 6/18 (33.3%) participants were able to perform at least one activity with Minimal or No Limitation (p=0.008 vs. baseline). At five years, 14/18 (77.8%) participants were able to perform one activity with Minimal or No Limitation (p<0.0001 vs. baseline) (Figure 4). 

Radiographic analysis

**Figure 5:**
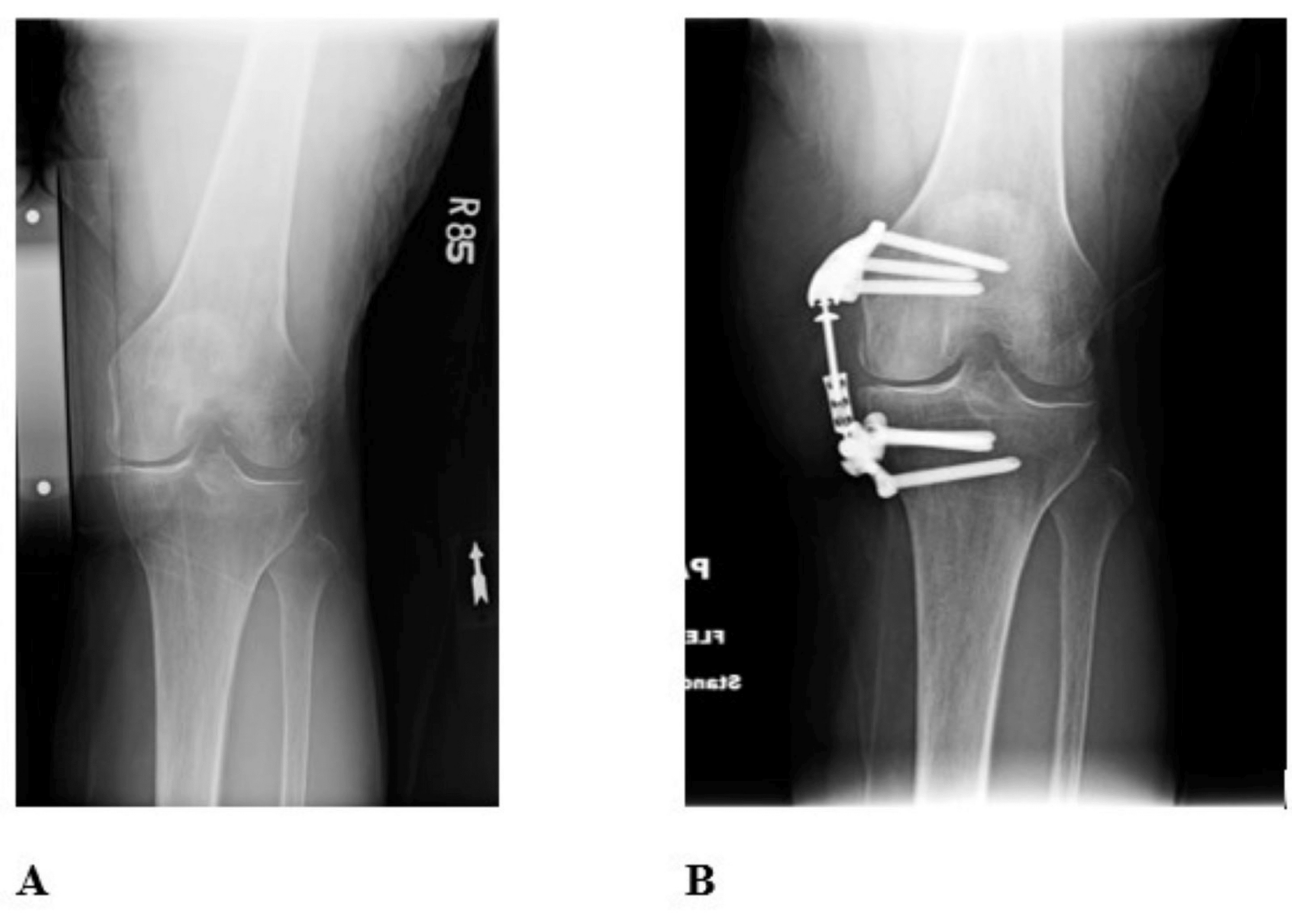
Preoperative posteroanterior (PA) radiograph (A) compared with the five-year postoperative radiograph (B) Imaging indicates preservation of joint integrity, implant stability, and no signs of loosening or hardware failure at five years post procedure.

Radiographic analysis was available for 18/18 (100%) participants at the five-year follow-up. Implant stability was observed in 17/18 (94.4%) participants (Figure 5). Device integrity was compromised in one patient, where device disassembly was observed at 105 days post procedure.

Adverse events

**Figure 6:**
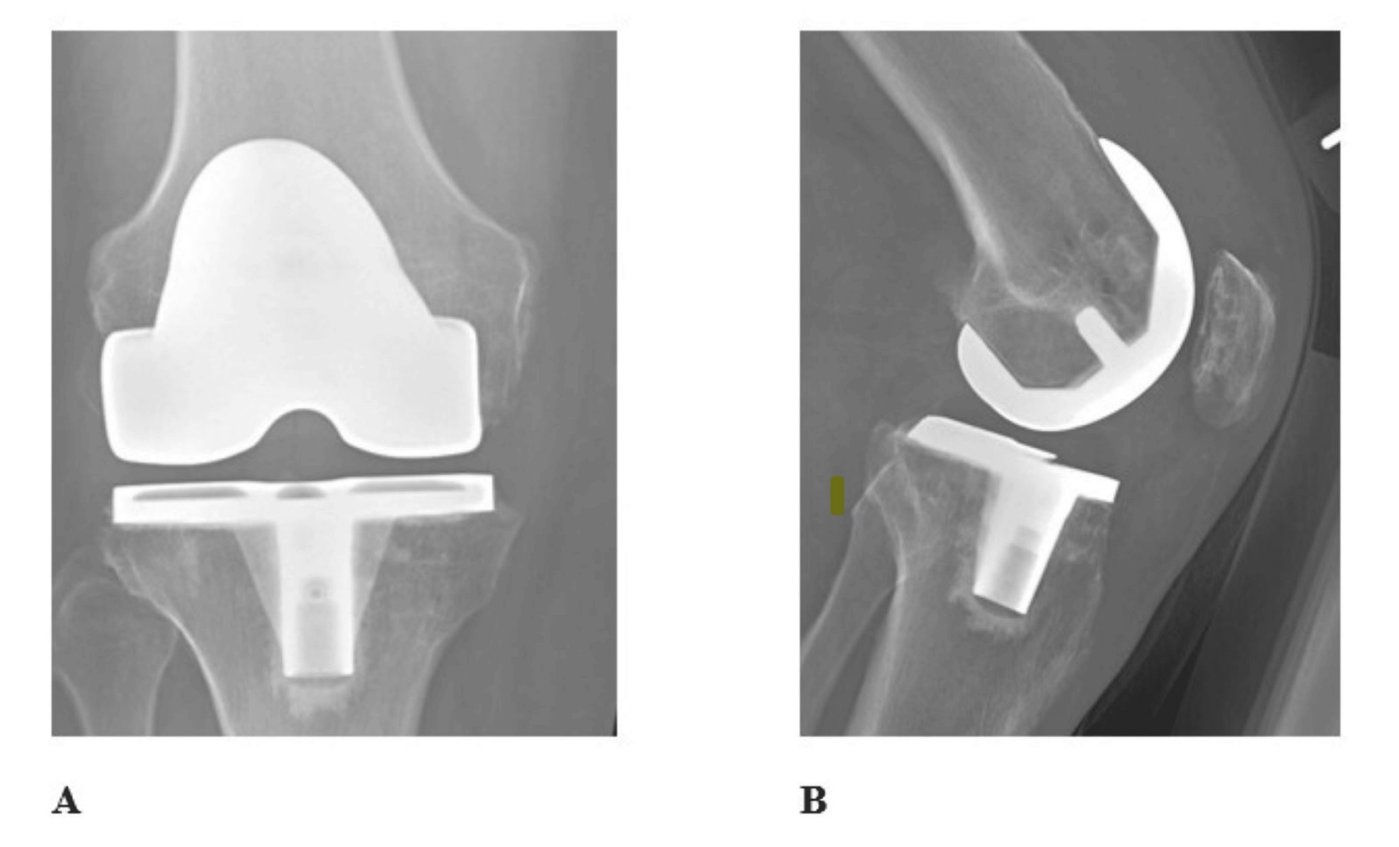
A participant underwent ISA removal after two years of follow-up and conversion to TKA at four years Five-year anteroposterior (A) and lateral (B) radiographs are presented above. TKA was performed as a primary arthroplasty procedure with no additional hardware and no evidence of implantable shock absorber-related complications. ISA: implantable shock absorber; TKA: total knee arthroplasty

An independent adjudication committee reported that 13/18 (72.2%) participants were free from SAEs at five years. One participant 1/18 (5.6%) reported the onset of discomfort prior to the two-year visit. Two participants, 2/18 (11.1%), reported pain, one with an onset during the initial six-month postoperative period and one with an onset prior to the two-year follow-up visit. Three participants 3/18 (16.7%) underwent conversion to TKA within the five-year follow-up period, due to advancement of arthritis in the knee. Figure 6 demonstrates post-TKA radiographs in one participant who underwent conversion.

Loss of implant integrity (absorber disassembly) was reported in one participant (1/18, 5.6%) at 105 days post procedure, with device removal at 113 days post procedure. Based on the criteria established in the Atlas and Calypso trials, this participant met the criteria for endpoint failure, although they did remain in the study for two additional years post removal, demonstrating a 55-point WOMAC pain change from baseline without conversion to arthroplasty.

## Discussion

OA of the knee is associated with a substantial disease burden, particularly among patients aged 65 and over, who constitute a large proportion of the affected population. Patients who are unsuitable for or unwilling to undergo TKA and do not respond to conservative care face a significant treatment gap, with few viable treatment alternatives. ISA technology has emerged as a solution to this treatment gap, with evidence from published trials derived from patients aged 27-80 at five years. This analysis focuses on the older quartile of this population (≥ 65 years at five years). In doing so, it demonstrates three principal findings: meaningful and sustained clinically validated improvements in pain and function, a robust safety profile, and preservation of joint integrity.

Although arthroplasty is one standard treatment for knee OA patients over age 65, it does not uniformly address all patients' needs or desired outcomes [31]. Older patients with mild-to-moderate disease are associated with poorer outcomes following arthroplasty, and a substantial proportion of older patients - up to 27% by some estimates - are unwilling to undergo TKA [19], thus limiting treatment options. Moreover, as life expectancy grows, patients have higher expectations for activity into their seventh, eighth, and ninth decades of life and are increasingly unwilling to accept activity-related limitations [32,33]. Given this climate, the need for effective treatment options is clear.

In our study, an ISA was associated with significant improvements in both pain and function in patients aged 65 and over, improvements that were maintained through five years of follow-up. Indeed, at five years post surgery, 13/14 (93%) patients aged 65 or older reported clinically meaningful improvements in pain, while 11/14 (79%) reported meaningful improvements in function. Overall, we observed a 70% reduction in pain and a 64% improvement in function five years post procedure. Additionally, 35/54 (65%) desired physical activities could be completed with minimal or no limitation five years after the procedure. Importantly, although the ISA has shown similar results in younger patient populations (success rates of 86.5% [22] and improvements in pain and function scores of 71% and 69%, respectively [23]), this is the first evidence of such improvements in an older patient population. The ability to achieve significant improvements in pain and function, and to maintain that improvement through five years without setbacks, is crucial, especially in an older population. Despite the absence of reimbursement barriers for non-TKA procedures, surgeons may favor arthroplasty in patients aged over 65, simply due to their age and potential for rapid disease progression. The data from our study provide evidence of long-term, sustained improvement in this population without the need for arthroplasty. This provides patients and surgeons with an additional treatment option and a means to potentially defer or delay arthroplasty, an important consideration for more than one in four patients who prefer to avoid this major surgical procedure [19]. The results here suggest that age should not be a limitation to the potential success of the ISA and that well-chosen patients age 65 or older can expect positive and long-lasting results from the use of an ISA.

In a previous study, Gomoll et al. observed a 90.6% survival rate at three years post procedure, with patients remaining free from arthroplasty or HTO during that period [23]. Pareek et al. showed similar results for one- and two-year follow-ups, with 100% of patients free from arthroplasty at each time point [26]. At five-year follow-up, the rate of conversion to arthroplasty remained consistent with prior reports, with 83% free from arthroplasty conversion at five years, with 94% of participants demonstrating implant stability. These results add to the growing body of evidence supporting the joint-preserving nature of the ISA. Important in our findings are not only the additional evidence of long-term stability with an ISA but also the confirmation of that stability in an older adult population, to supplement previous studies that focused on younger cohorts. Equally important, particularly for older adults, is that delaying arthroplasty reduces the lifetime risk of revision, which relieves the burden of repeated major surgeries from patients and reduces the associated costs for the healthcare system. A recent study from the New Zealand Joint Registry calculated the lifetime risk of revision following TKA at 22.4% in patients between age 46 and 50 years and determined that the risk decreased linearly to as low as 1.15% in patients aged 90 and over [34]. The revision risk in patients aged 71-75 years (5.87%) was over half that of patients aged 60-65 (11.2%), with patients over 80 seeing their risk decrease to less than 3%. As such, delaying TKA could result in a shift in patients to a lower revision risk range. Our findings suggest that the ISA may be an avenue to promoting this shift and thus reducing the need for multiple invasive procedures, which benefits not only the patient but may also help alleviate the economic burden on healthcare systems [35-37].

Adverse events were reported infrequently in our study, with 13/18 (72%) participants remaining free from SAEs during the five-year follow-up period, as adjudicated by an independent Clinical Events Committee. At five years, three participants experienced disease progression sufficient to necessitate conversion to TKA, and one participant reported device failure. Our results align with those from similar studies, including those enrolling younger populations, where AE rates of 16% have been reported [22]. As with other studies [22,23], no surgical site infections were reported in our study, and post-operative pain was reported rarely. Indeed, in several studies of the ISA, especially those that compared ISA with HTO, pain was consistently reported in a greater proportion in the HTO group than the ISA group [22,23,38]. This lack of post-surgical pain removes an immediate obstacle to mobility and may help to speed recovery.

Our study is not without limitations. The relatively small sample size may be considered a limitation, as could the non-randomized nature of the study. These concerns, however, are offset by the fact that the participants in our study were recruited as part of larger studies and subject to stringent and robust eligibility criteria. As such, the small sample is offset by the methodological strength of the trial enrollment process, which also minimizes the potential for selection bias. The lack of a control group is also a limitation; however, given the long follow-up period, the comparison-to-baseline analysis provides important insight into the patient experience with the ISA over time. Future studies, though, would benefit from a control group. Finally, the combining of participants from two separate studies may be considered a limitation; however, the eligibility criteria for these studies were well aligned, such that all participants included in this analysis were subject to analogous eligibility criteria, allowing the creation of a single cohort of participants suitable for analysis. While these limitations may affect the generalizability of the results somewhat, the results are the first analysis in this specific age group and provide an early indication of the treatment effectiveness in this population. Despite this, more research is needed.

## Conclusions

Our study demonstrated that the ISA is a viable intervention for a selected population of patients with symptomatic knee OA who have failed non-surgical care. In this challenging patient population, the ISA was associated with significant and long-term improvements in pain and function while preserving the joint and maintaining the option for future arthroplasty if needed. As the demand for patient-centered and stage-appropriate solutions grows, interventions that offer options that align with the dual goals of improving immediate quality of life while preserving joint integrity are necessary. For patients suffering in the treatment gap between the limited effectiveness of non-surgical approaches and the finality of arthroplasty, treatment with ISA offers a viable option.
